# Overexpression of Ubiquitin Specific Protease 44 (USP44) Induces Chromosomal Instability and Is Frequently Observed in Human T-Cell Leukemia

**DOI:** 10.1371/journal.pone.0023389

**Published:** 2011-08-11

**Authors:** Ying Zhang, Jan van Deursen, Paul J. Galardy

**Affiliations:** 1 Department of Pediatrics and Adolescent Medicine, Mayo Clinic, Rochester, Minnesota, United States of America; 2 Department of Biochemistry and Molecular Biology, Mayo Clinic, Rochester, Minnesota, United States of America; Virginia Tech, United States of America

## Abstract

Cdc20-anaphase promoting complex/cyclosome (Cdc20-APC/C) E3 ubiquitin ligase activity is essential for orderly mitotic progression. The deubiqituinase USP44 was identified as a key regulator of APC/C and has been proposed to suppress Cdc20-APC/C activity by maintaining its association with the inhibitory protein Mad2 until all chromosomes are properly attached to the mitotic spindle. However, this notion has been challenged by data in which a lysine-less mutant of Cdc20 leads to premature anaphase, suggesting that it's ubiquitination is not required for APC/C activation. To further evaluate its role in checkpoint function and chromosome instability, we studied the consequences of over-expression of mouse Usp44 in non-transformed murine embryonic fibroblasts. Here we show that cells with high Usp44 are prone to chromosome segregation errors and aneuploidization. We find that high Usp44 promotes association of Mad2 with Cdc20 and reinforces the mitotic checkpoint. Surprisingly, the APC/C-Cdc20 substrate cyclin B1 is stabilized in G2 when Usp44 is over-expressed, but is degraded with normal kinetics once cells enter mitosis. Furthermore, we show that USP44 expression is elevated in subset of T-cell leukemias. These data are consistent with an important role for USP44 in regulating Cdc20-APC/C activity and suggest that high levels of this enzyme may contribute to the pathogenesis of T-ALL.

## Introduction

The cells of a majority of human cancers carry abnormal numbers of chromosomes, a condition known as aneuploidy [1]. Although the role of aneuploidy in the genesis of cancer has been long debated, recent data from mice strongly suggest that, at least in some instances, aneuploidy does cause cancer [2], [3], [4]. For most tumor types, the mechanisms leading to aneuploidy are unknown. In order to preserve genomic integrity, cells must ensure the accurate and timely segregation of chromosomes to daughter cells in mitosis. A wide array of gene products are required to maintain high fidelity chromosome segregation including those involved in chromosome condensation, spindle assembly, microtubule attachment to chromosomes, mitotic checkpoint control, sister chromatid separation, and others [5]. A complex pathway known as the spindle assembly checkpoint, or mitotic checkpoint, ensures that the transition to anaphase is delayed until all chromosome kinetochores are properly attached to the mitotic spindle [6], [7], [8]. At the heart of this mechanism is a large multi-subunit ubiquitin E3 ligase known as the anaphase promoting complex/cyclosome (APC/C) that targets the separase inhibitors securin and cyclin B for proteasomal degradation [9], [10]. The degradation of these proteins leads to the activation of separase that cleaves cohesin rings that join sister chromatids, resulting in anaphase. To prevent untimely anaphase onset, the activity of APC/C is tightly regulated through the binding of an inhibitory complex consisting of the checkpoint components Mad2, BubR1, and Bub3 (known as the Mitotic Checkpoint Complex, or MCC) to the APC/C co-activator molecule Cdc20 [11], [12], [13]. Upon attachment of spindle microtubules to all chromosome kinetochores, the MCC dissociates from the APC^Cdc20^ and the APC becomes active.

Recently, two RNAi-based functional genetic screens were performed in order to identify novel gene products involved in the mitotic checkpoint [14], [15]. In both studies, depletion of the de-ubiquitinase USP44 led to a bypass of the mitotic checkpoint. According to the proposed model, checkpoint silencing requires the ubiquitin conjugating enzyme UbcH10 to polyubiquitinate Cdc20, leading to the dissociation of MCC components, activation of APC/C ligase activity and anaphase onset [16]. In opposition to the activity of UbcH10, USP44 is thought to restrain APC/C activity by de-ubiquitinating Cdc20, thus preventing MCC dissociation and untimely anaphase onset. This model, however, has recently been challenged by data in which a lysine-less mutant of Cdc20 was able to properly function as an APC/C activator. Rather than arresting cells in metaphase due to an inability to silence the checkpoint, this lysine-less mutant actually hastened premature mitotic exit in nocodazole arrested cells [17]. Therefore, the mechanism by which UbcH10 and USP44 regulate checkpoint signaling is unclear.

To address issues raised by previous RNAi studies, we have studied the consequences of over-expression of Usp44 in non-transformed mouse fibroblasts. As was observed in cells depleted of USP44, we observe increased levels to grossly disrupt normal chromosome segregation, leading to aneuploidy. These changes are accompanied by functional and biochemical evidence of reduced Cdc20-APC/C activity, with the substrate cyclin B1 stabilized in G2, and early mitosis. These observations suggest that USP44 is an inhibitor of APC/C activity. Lastly, we show that levels of USP44 are highly elevated in human T-cell acute lymphoblastic leukemia, suggesting a role for these molecular defects in the pathogenesis of this disease.

## Results

### USP44 over-expression leads to whole chromosome instability (W-CIN)

**Figure 1:**
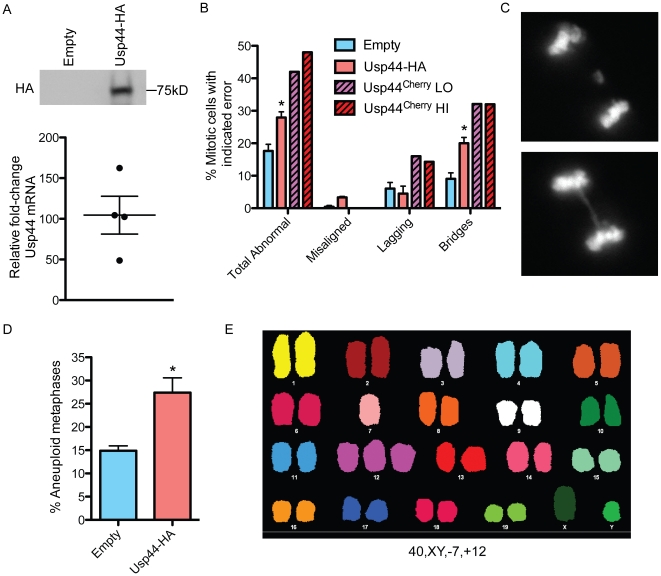
Expression of Usp44 leads to chromosome missegregation and aneuploidy. (a) Expression of Usp44-HA in MEFs as seen by immunoblotting (upper) or quantitative real-time (qRT)-PCR (lower). Four independent MEF lines were transduced with Usp44-HA or Usp44Cherry followed by TaqMan qRT-PCR. Expression was normalized to GAPDH, and fold-change was calculated using the ΔCt method. (b) MEFs stably transduced with either empty vector (n = 78 cells), Usp44-HA (n = 106 cells), or Usp44^Cherry^ (n = 53 total) were analyzed by live-cell microscopy through the indicated numbers of un-perturbed cell divisions. Chromosomes were visualized by transduction with lentivirus encoding histone H2B fused with yellow fluorescent protein (H2B-YFP). The results depict the average and standard error from three-independent MEF lines. (c) Example of a cell with a lagging chromosome (upper) and anaphase bridge (lower). (d) Karyotype analysis of low passage MEFs. Stable transduction with the indicated lentivirus was performed at passage 2. N (metaphases)  = 175 (Empty vector control) and 223 (Usp44-HA) from a total of four independent MEF lines per genotype. (e) Representative spectral karyotype (SKY) of a cell expressing Usp44-HA. Note this cell has a normal chromosome number (40) but has a trisomy of chromosome 12 and monosomy of chromosome 7. * p<0.05 calculated with the unpairted t-test. Error bars represent the SEM.

To examine the impact of excess Usp44 on mitotic chromosome segregation, we used live-cell microscopy to follow MEFs transduced with lentivirus encoding either empty, or Usp44-HA, as they progress through mitosis. Chromosomes were visualized through the expression of histone H2B-YFP. When compared with control cells, we observed a significant increase in chromosome missegregation in MEFs expressing Usp44-HA (Figs. 1A, B, C; 31.3% v. 17.6%, p<0.001) with the predominant defect being anaphase bridges. The ability of these mitotic errors to produce aneuploidy was determined through chromosome counts. Compared with cells transduced with empty lentivirus, those expressing Usp44-HA had a significant increase in aneuploidy (27.4% v. 14.9%; p<0.05, Fig. 1D).

As these data come from a polyclonal pool of drug-resistant MEFs, there is likely to be significant variation in the level of Usp44 over-expression. To determine if mitotic errors vary with the level of expression, we transduced MEFs with a fusion between Usp44 and the fluorescent protein mCherry (Usp44^Cherry^). We conducted live-cell imaging experiments and grouped cells by the level of expression – with those having dim expression (Usp44^Cherry^LO) and others with bright expression (Usp44^Cherry^HI). The rate of chromosome missegregation was increased in both groups compared with the Usp44-HA pool, with 42% abnormalities in the Usp44^Cherry^LO group, and 48% in the Usp44^Cherry^HI group, with the predominant abnormality being chromosome bridging. This suggests that the data from cells expressing Usp44-HA may underestimate the mitotic defects resulting from excess Usp44.

To further characterize the nature of the chromosome instability resulting from excess Usp44, we performed spectral karyotyping (SKY) on primary MEFs. Expression of wild-type Usp44 was associated with a substantial increase in whole chromosome losses or gains compared with cells transduced with empty virus (Fig. 1E). An average of 3.8 chromosomes were lost or gained per cell expressing Usp44-HA and 2 per cell transduced with empty virus. There were no clonal rearrangements seen, though a small number (10-15%) of cells from both genotypes harbored chromosomes with translocations. Taken together, these data demonstrate that excess Usp44 leads to chromosome segregation errors, and aneuploidy.

Despite several attempts (five different immunogens in ten rabbits), we were unsuccessful in raising a polyclonal antibody suitable for the detection of endogenous mouse or human USP44 by western blot, nor did any of the commercially available antibodies recognize over-expressed human or mouse Usp44. Thus, it impossible to determine the degree of protein over-expression. We therefore determined the abundance of Usp44 mRNA by quantitative real-time (qRT)-PCR in a panel of 4 MEF lines used in the experiments reported here. In these cells, we detected an average 95-fold (range 48-162; Fig. 1A) increased Usp44 mRNA from asynchronous cell cultures. The chromosome missegregation and aneuploidy did not vary significantly between lower or higher over-expressers (data not shown). As is discussed in detail below, we believe Usp44 is subject to extensive post-translational regulation via proteasome degradation, making a direct correlation between mRNA and protein difficult to establish.

### Over-expression of Usp44 reduces mitotic slippage in nocodazole

**Figure 2:**
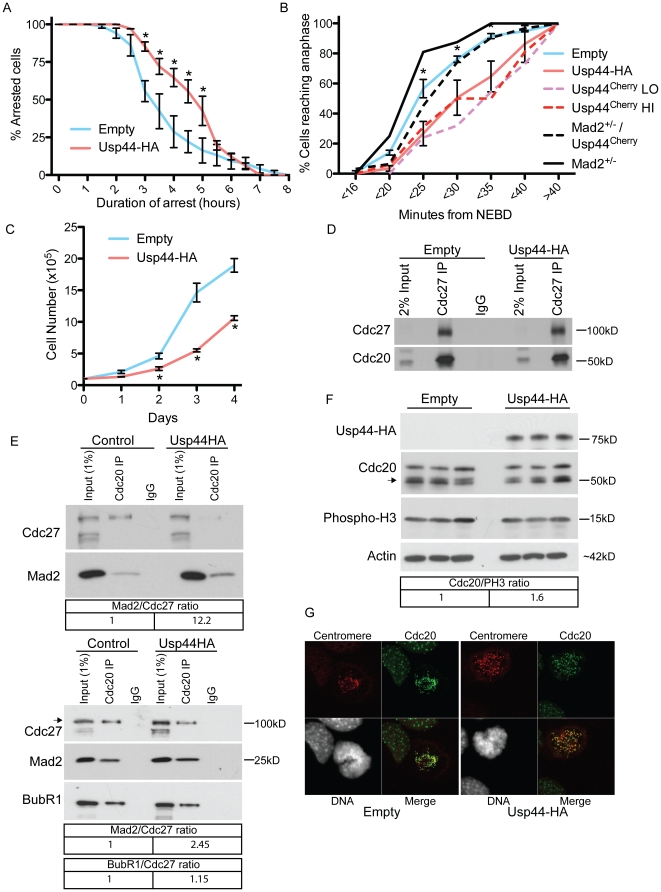
Usp44 expression leads to reduced mitotic slippage. (a) The duration of mitotic arrest in nocodazole was determined for MEFs transduced with either empty vector or Usp44-HA (n = 40–60 cells each). Chromosomes were visualized by prior transduction with lentivirus expressing H2B-YFP. After the addition of nocodazole, cells entering mitosis (for up to 20 minutes) were marked and followed by time lapse imaging for over 9 hours. The duration of arrest was defined as the time from nuclear envelope breakdown (NEBD) to de-condensation of chromosomes and nuclear reformation. (b) The duration of mitosis was determined by live-cell microscopy of MEFs with the indicated constructs. All cells were transduced with H2B-YFP and the time from nuclear envelope breakdown (NEBD) to anaphase onset was determined. N = 79 cells with control, 84 with Usp44-HA, 21 Usp44^Cherry^LO, 20 Usp44^Cherry^HI. p-values were calculated using the 2-way ANOVA. (c) The growth of cells was determined for three independent MEF lines transduced with either empty vector, or Usp44-HA. Viable cells were counted daily in triplicate using trypan blue exclusion. (d) Immunoprecipitation of Cdc27 from MEFs with or without over-expressed Usp44-HA. Precipitated material was immunoblotted with the indicated antibodies. (e) Immunoprecipitation of Cdc20 from MEFs with or without over-expressed Usp44-HA. Two results out of six independent experiments are shown. Relative amounts of Mad2 and BubR1, normalized to the mount of Cdc27 retrieved, are shown. (f) Three independent MEF lines were transduced with the indicated constructs and mitotic cells isolated by shake-off in the presence of nocodazole. Extracts were probed with the indicated antibodies. (g) Cdc20 localization was assessed by confocal microscopy on MEF cultures transduced with the indicated constructs. Images are representative of the results seen in three independent MEF lines. Relative amounts of Cdc20, normalized to the amount of PH3, are shown. * Unless otherwise noted, p<0.05 for each indicated time point calculated with an unpaired t-test. Error bars represent the SEM.

When the mitotic checkpoint is activated through the incubation with either nocodazole or taxol, cells eventually die, or exit mitosis without cytokinesis, a process called mitotic slippage. This event has been shown to depend on cyclin B1 degradation [18], and occurs more quickly in cells harboring defects in checkpoint signaling [19], [20], [21]. To examine whether excess Usp44 alters mitotic arrest kinetics, we transduced MEFs with either empty or Usp44-HA encoding lentivirus and incubated them in the presence of the spindle poison nocodazole. We examined the ability of single cells to maintain a mitotic arrest using live cell microscopy. Following the addition of nocodazole, cells entering mitosis were marked and followed over 12 hours. The duration of arrest was determined as the time from nuclear envelope breakdown to chromosome decondensation (mitotic exit without cytokinesis). In control cells, 50% of cells have exited mitosis by between 3 and 3.5 hours (Fig. 2A). In the presence of excess Usp44, mitotic arrest was prolonged with the 50% exit time being 4.5–5 hours (140% control; p<0.05). This demonstrates that mitotic slippage is reduced in the presence of excess Usp44. As cells rarely require this length of time to complete mitosis under physiological conditions, we asked whether Usp44 expression influenced the duration of mitosis in the absence of spindle poisons. To measure this, we observed cells by live cell imaging and calculated the interval between nuclear envelope breakdown and anaphase onset. Compared with control MEFs, those expressing Usp44-HA had a significant delay in reaching anaphase with the median time increased from between 21–25 minutes for control to between 26–30 minutes for Usp44-HA (Fig. 2B). Consistent with this observation, we observe a slower growth rate for cells expressing excess Usp44 compared with control (Fig. 2C). To determine if the mitotic timing varies with the level of Usp44 expression, we conducted live-cell imaging experiments and again compared Usp44^Cherry^LO and Usp44^Cherry^HI cells. There was no difference in the duration of mitosis between cells in the LO or HI groups, with both groups delayed when compared with empty vector controls. To determine if this delay in mitotic exit is dependent on mitotic checkpoint signaling, we expressed Usp44^Cherry^ in MEFs heterozygous for the essential APC^Cdc20^ inhibitor, Mad2, and again performed live-cell microscopy. Consistent with a role for the checkpoint, we found that the delay imparted by excess Usp44 was entirely eliminated in Mad2^+/−^/Usp44^Cherry^ MEFs. Consistent with delayed mitotic exit, we observe a slower growth rate for cells expressing excess Usp44 compared with control (Fig. 2C).

The mitotic checkpoint restrains anaphase onset through the association of the MCC components BubR1, Mad2, and Bub3, with the APC^Cdc20^. To examine the effect of excess Usp44 on the assembly of APC^Cdc20^ and the MCC on APC^Cdc20^, we performed co-immunoprecipitations from control or Usp44 expressing cells. We first asked whether the assembly of APC^Cdc20^ was altered in the presence of excess Usp44. We immunoprecipitated the APC/C component Cdc27 and probed for Cdc20 to assess whether Usp44 produced changes in the association of Cdc20 with the core subunits of APC/C. In several experiments, we observed no impact of Usp44 expression on the assembly of APC^Cdc20^ (Fig. 2D). We then asked whether Usp44 changed the assembly of the MCC on APC^Cdc20^ by precipitating Cdc20 from mitotic cells and probing for Mad2. We were unable to determine the efficiency of Cdc20 immunoprecipitation due to antibody incompatibility. Given our results showing that Usp44 does not impact the association of Cdc20 with Cdc27, we used the level of Cdc27 as a surrogate for the equality of Cdc20 recovery. In repeated experiments, we observed a modest but reproducible increase in the recovery of Mad2 with Cdc20, suggesting that excess Usp44 increases the association of Mad2 with Cdc20 (Fig. 2E). The effect on Mad2 association appears specific, as a similar accumulation is not seen with BubR1.

In light of the recent reports demonstrating significant turnover of Cdc20 in mitosis, we next examined whether excess Usp44 altered the level or localization of Cdc20. Three independent mitotic MEF lines were transduced with either empty vector, or Usp44-HA and were subjected to western blotting for Cdc20. No substantial changes in Cdc20 level were observed (Fig. 2F). Consistent with this result, we observed no differences in the localization of Cdc20 to the kinetochore in mitotic cells transduced with control, or Usp44-HA (Fig. 2G). In aggregate, these data indicate that excess Usp44 leads to a reinforcement of the mitotic checkpoint, likely by increasing the association of Mad2 with Cdc20.

### Usp44 over-expression leads to increased Cyclin B prior to mitosis

**Figure 3:**
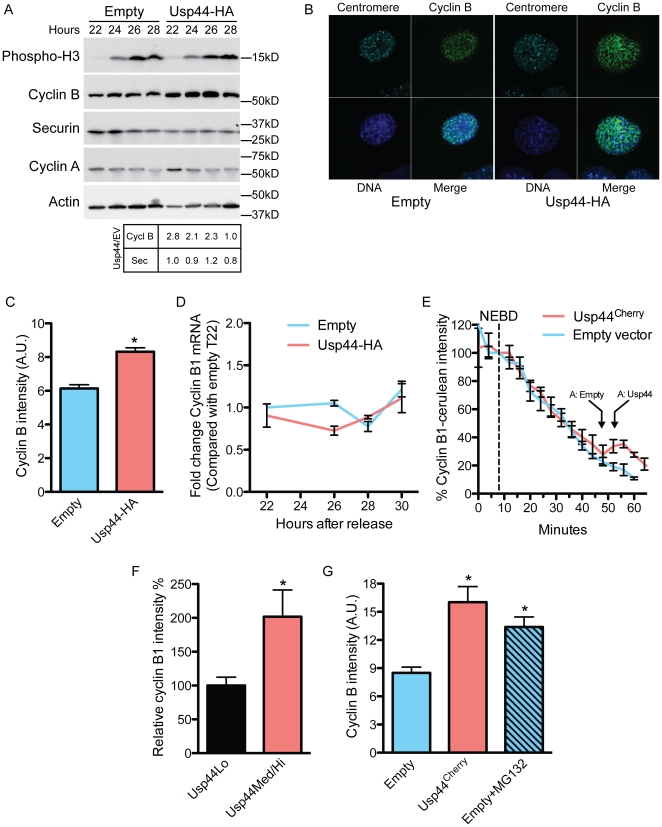
Excess Usp44 leads to increased cyclin B in early mitosis. (a) Cells were synchronized in G1/G0 by serum starvation and were then released. Nocodazole was added 23 hours after release. Samples were collected at the indicated times and were immunoblotted with the indicated antibodies. Results are representative of at least 3 independent experiments. (b) Immunofluorescence imaging of cells transduced with empty lentivirus or Usp44-HA using the indicated antibodies. The stage of mitosis was determined by DNA morphology. (c) Quantitation of cyclin B levels from (a) using imageJ. Ten cells from each of three independent MEF lines (total 30 cells per condition per stage) were analyzed. (d) Cyclin B1 mRNA was measured using qRT-PCR. Cells were synchronized as in (a) and harvested at the indicated times. (e) MEFs were stably transduced with the indicated construct and were then transfected with a construct encoding a fusion between cyclin B1 and Cerulean (CyclinB1^Cerulean^). Cells were monitored by live-cell microscopy. Images were obtained every 4 minutes and quantified with imageJ. Values were normalized such that the level at nuclear envelope breakdown (NEBD) was set at 100%. The arrow with “A:” refers to the average time of anaphase observed in the cells in each condition. (f) MEFs were transduced with the indicated constructs and were fixed and stained to detect Usp44^Cherry^, cyclin B1, and DNA. The levels of Usp44^Cherry^ and cyclin B1 were quantitated in G2 cells (n = 11–19 cells each) using imageJ. (g) MEFs transduced with empty vector were treated with MG132 for 1 hour prior to fixation. The amount of cyclin B1 was determined in G2 or early prophase using imageJ in comparison to untreated, or Usp44^Cherry^ transduced MEFs. Graph represents the average of 20 cells in each group from three independent experiments. * p<0.05 calculated with an unpaired t-test.

To examine the impact of excess Usp44 on APC/C activity, we determined the level of a subset of APC/C substrates by immunoblotting. Immortalized MEFs expressing Usp44-HA were arrested in G1 by serum starvation, followed by release into serum containing medium. Cells were arrested in mitosis by the addition of nocodazole 23 hours following the release into serum. As determined by immunoblotting for phosphorylated histone H3 (PH 3), cells began to enter mitosis within 24 hours following release. Compared with control, Usp44-HA expression led to an increase in the levels of cyclin B at all time points, suggesting reduced APC/C activity (Fig. 3A). Notably, we observe high levels of cyclin B at times where there are low levels of PH 3, suggesting that cyclin B stabilization may occur prior to mitosis. In contrast to cyclin B, we observed no effect of Usp44 on the levels of securin. To further investigate the effect of Usp44 on the levels of cyclin B, we examined its level by confocal immunofluorescence in unchallenged cells. MEFs were stably transduced with Usp44-HA and compared with control empty virus transduced cells. Cells were co-stained for PH 3 and DAPI, and the levels of cyclin B were quantified using ImageJ software (http://rsbweb.nih.gov/ij/). In early mitosis, we observed a significant increase in cyclin B1 levels compared with control (Figs. 3B, C). In subsequent phases of mitosis, there was no difference in cyclin B1 across all groups. To exclude that this increase was due to transcriptional changes, we performed qRT-PCR at these time points and found no changes in cyclin B1 mRNA (Fig. 3D).

High levels of cyclin B in prophase may be the result of elevated levels of the protein at mitotic entry, but also due to delays in the degradation in mitosis, or both. We first asked whether the kinetics of cyclin B degradation in mitosis is altered by exogenous Usp44. To accomplish this, we introduced a reporter construct consisting of cyclin B1 fused with the fluorescent protein Cerulean (cyclin-B1^Cerulean^) into MEFs stably transduced with either empty virus or Usp44^Cherry^ (along with H2B-YFP) and then followed cells by live-cell microscopy. We marked cells in G2, either by centrosome association of cyclin B1, or by recognizing the early stages of chromosome condensation, and followed them through mitosis. As there is no cycloheximide present, the amount of cyclin-B1^Cerulean^ present is the balance of new synthesis and degradation. As has been reported by others using mouse cells, we observe that cyclin B1 levels gradually decline following nuclear envelope breakdown (Fig. 3E) [22]. This differs from human cells where the level of cyclin B1 has been shown to remain stable from nuclear envelope breakdown until just prior to anaphase onset, at which point the levels decline abruptly.[23] We found no differences in the rate of cyclin-B1^Cerulean^ degradation in Usp44^Cherry^ positive cells compared with control. We next asked whether levels of cyclin B1 are elevated prior to entering mitosis. MEFs, expressing Usp44^Cherry^ were stained for mCherry and cyclin B1 and were classified as Usp44 HI or LO based on quantitation using imageJ. Late G2 cells were selected based on very early chromosome condensation along with cytoplasmic cyclin B1. Compared with cells with low or absent expression, cells with high levels of Usp44^Cherry^ have a significant increase in cyclin B1 in G2 consistent with a stabilizing effect prior to mitosis (Fig. 3F). To confirm that the differences in cyclin B1 in G2 and prophase are due to variances in proteasomal degradation, we examined the level of cyclin B1 in MEFs treated for one hour with MG132. Compared with those transduced with empty lentivirus, we again observed a significant increase in cyclin B1 at G2 and prophase in cells expressing Usp44^Cherry^ (Fig. 3G). This difference was eliminated by pre-treatment of control cells with MG132, suggesting that at these phases, cyclin B1 is subjected to proteasomal degradation. Taken together, these data suggest that exogenous Usp44 leads to increased cyclin B1 in G2 and prophase. Given the described role for Usp44 in regulating APC/C activation, and our finding of increased Mad2 complexed with Cdc20, we conclude that Usp44 affects cyclin B1 levels by inhibiting APC activity prior to the onset of mitosis.

### Usp44 is a nuclear protein that undergoes ubiquitin-dependent degradation prior to mitosis

**Figure 4:**
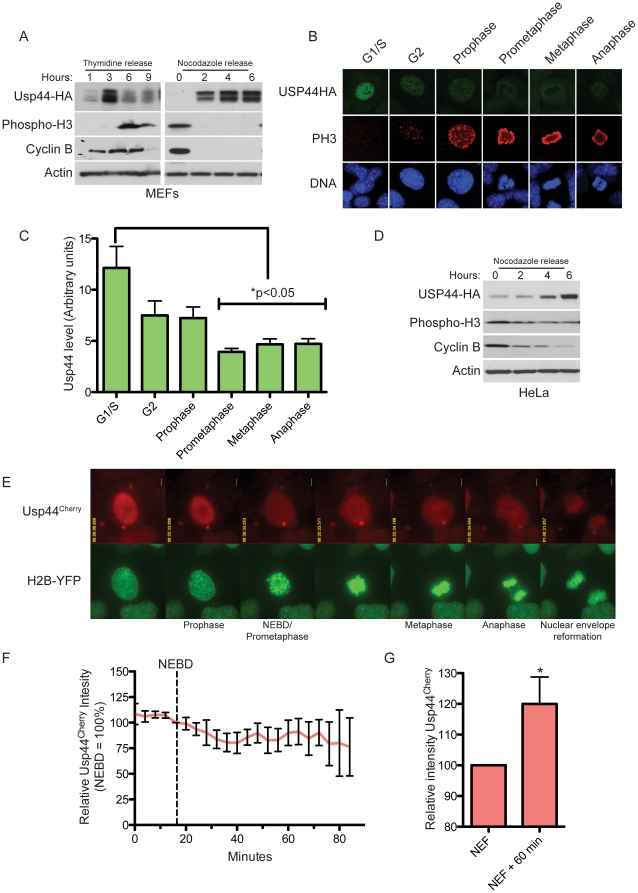
Usp44 undergoes degradation prior to mitosis. (a) Immunoblot of cells expressing Usp44-HA previously synchronized with double thymidine (DT) or nocodazole (Noc). Samples were harvested at the indicated times after release, and were immunoblotted with the indicated antibodies. (b) Immunofluorescence images of Usp44-HA in different cell cycle phases. G1/S, G2, and prophase cells were distinguished based on the patterns of chromosome condensation and phospho histone H3 staining (absent in G1/S, punctate in G2 and prophase). Staining performed with the indicated antibodies. (c) quantitation of immunofluorescence from (b). Levels of HA immunoreactivity were determined using imageJ (n = 8 cells each). (d). HeLa cells were analyzed as in (a). (e) Time-lapse images of a representative mitotic MEF expressing Usp44^Cherry^ and H2B-YFP. Relative times are indicated. (f) Quantitation of Usp44^Cherry^ fluorescence (n = 10) in MEFs monitored by live-cell microscopy from late G2 through anaphase. The levels of mCherry fluorescence were determined using imageJ. (g). MEFs expressing Usp44^Cherry^ (n = 9) were monitored by live cell microscopy and the levels of mCherry fluorescence was measured at nuclear envelope re-formation (NEF) and at NEF+60 minutes. * p<0.05 calculated with an unpaired t-test. Error bars represent the SEM.

In order to better understand its role in mitosis, we tested if Usp44 is regulated in a cell cycle specific fashion. To accomplish this, we examined the expression pattern of epitope tagged Usp44 in stably transduced MEFs. We first synchronized cells with double thymidine followed by release into complete medium to examine levels of Usp44-HA by immunoblotting as the cells proceeded through an unchallenged mitosis. Surprisingly, the levels of Usp44-HA increased after release from double thymidine and peaked just prior to mitosis (as determined by levels of phosphorylated histone H3 (Ser10; PH 3); Fig. 4A). There was an abrupt decline in Usp44-HA level once cells were in mitosis. To confirm these findings, we obtained mitotic cells by shake-off from nocodazole-arrested cultures and released them into complete medium lacking nocodazole. We again observed relatively low levels of Usp44-HA in mitotic cells (shake-off time 0 hours) and a rapid increase after mitotic exit (as judged by the loss of cyclin B) (Fig. 4A). Similar results were obtained when we analyzed human USP44-HA expressed in HeLa cells (Figure 4D). To exclude artifacts that may result from synchronization techniques, we next examined the level of Usp44-HA in asynchronous unchallenged cells with confocal immunofluorescent microscopy. Cells were co-stained with phospho-histone H3 and DAPI to assist in determining cell cycle phase. Usp44-HA levels peaked in G1/S (as determined by negative phospho-histone H3 and interphase DNA pattern) and were already reduced in G2 (speckled phospho-histone H3 and interphase DNA pattern; Figs. 4B, C). Levels of Usp44-HA subsequently decreased further as cells entered mitosis (diffuse phospho-histone H3 with condensed chromosomes).

To gain further insight into its regulation, we tracked MEFs expressing Usp44 Cherry and H2B-YFP through mitosis using live-cell microscopy. The Usp44^Cherry^ fusion protein localized predominantly to the nucleus, with scattered signal in the cytoplasm in a punctate or vesicular pattern near the nucleus (Fig. 4E). As cells entered mitosis, there was a sudden redistribution of the mCherry signal into the cytosol that occurs prior to the onset of prometaphase. This likely represents nuclear envelope breakdown. As predicted by the immunostaining data, levels of Usp44^Cherry^ declined slightly from just before mitotic entry through to anaphase (Fig. 4E, F). Following anaphase, Usp44^Cherry^ regained a tight association with the chromosomes suggesting reformation of the nuclear envelope. Starting at this point, there was a significant increase in levels of Usp44^Cherry^ over the subsequent 60 minutes, consistent with our immunoblot data (Fig. 4G). Taken together, these data support the conclusion that exogenous Usp44 peaks in interphase, with relatively low levels maintained throughout mitosis.

**Figure 5:**
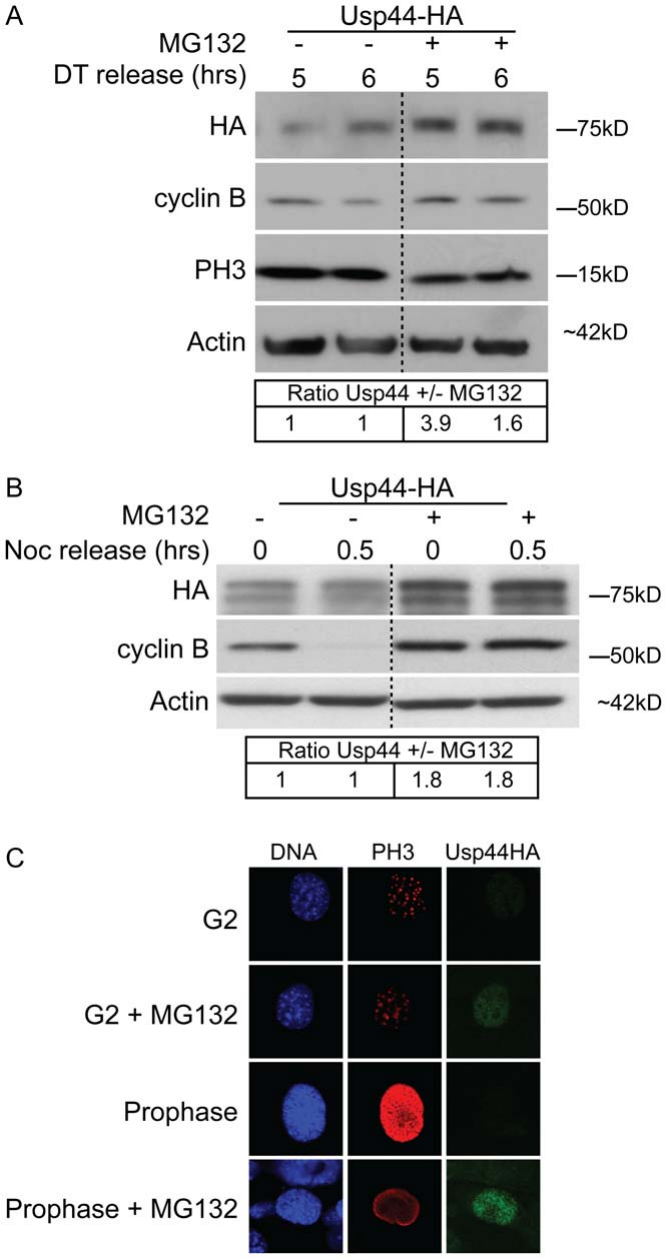
Usp44 is degraded by the proteasome. (a, b) Immunoblots of MEFs synchronized either by double-thymidine block (a) or mitotic shake-off (b), expressing Usp44-HA. Cells were incubated with MG132 3 hours after double-thymidine release or one hour prior to mitotic shake off. Relative quantitation was performed using imageJ. (c) Immunofluorescence images of cells expressing Usp44-HA treated (where indicated) with MG132 one hour prior to fixation.

Given that our exogenous constructs are under the constitutive control of a CMV promoter, we hypothesized that the cell cycle specific changes in Usp44-HA are due to post-translational events. USP44 was recently found to be modified with K48-linked poly-ubiquitin chains, suggesting that it may be subjected to proteasomal degradation [24]. We therefore investigated the nature of the decline in Usp44 through the use of the proteasome inhibitor MG132. Usp44-HA expressing MEFs were synchronized with double thymidine and released into complete medium as before, except the proteasome inhibitor MG132 was added 3 hours after the release to correspond to peak levels of Usp44-HA. We found that proteasome inhibition stabilized USP44 whereas in control cells Usp44-HA levels remained lower (Fig. 5A). Similarly, mitotic MEFs were incubated with nocodazole for 1 hour prior to release into complete medium lacking nocodazole. Following proteasome inhibition, Usp44 levels were significantly stabilized compared with control (Fig. 5B). Levels of USP44 in mitosis were also boosted by proteasome inhibition as seen by confocal immunofluorescent microscopy (Fig. 5C). We conclude that Usp44-HA is degraded by the proteasome prior to and during mitosis, and is rapidly re-synthesized upon mitotic exit.

### USP44 is frequently overexpressed in T-cell acute lymphoblastic leukemia (ALL)

**Figure 6:**
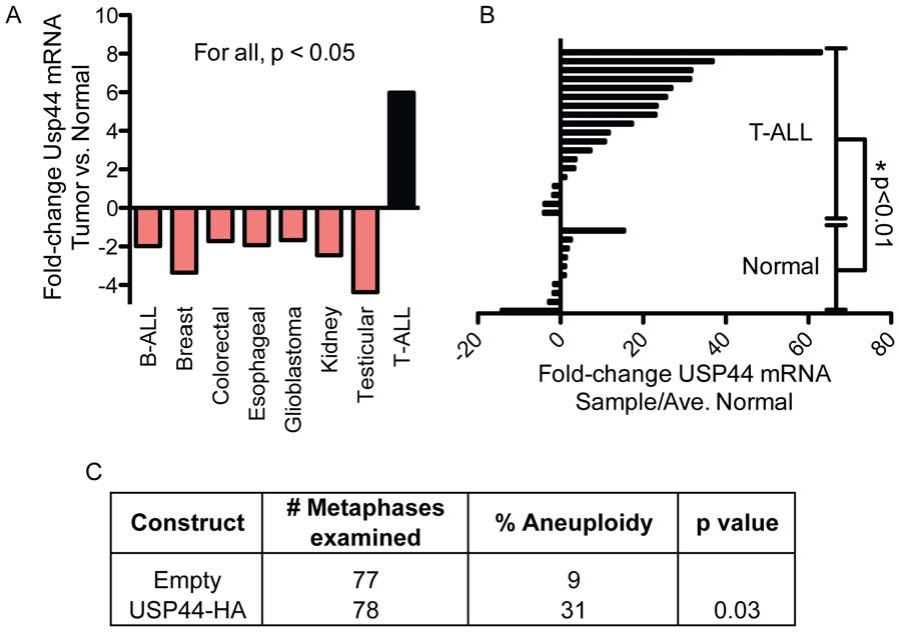
USP44 is over-expressed in T-cell acute lymphoblastic leukemia. (a) Relative USP44 mRNA expression in various cancer types versus matched normal tissue was collected from publicly available microarray studies (www.oncomine.org). For all studies shown, p<0.05 for alterations in USP44 mRNA. (b) USP44 mRNA was measured by TaqMan quantitative real-time PCR in a series of 24 samples of T-cell acute lymphoblastic leukemia compared with peripheral T-cells isolated from 10 healthy volunteers. The p value was calculated with an unpaired t-test comparing the mean fold-change of T-ALL to controls. (c) Human foreskin fibroblasts were transduced with the indicated constructs (two independent lines for each), cultured for 5-passages, and then analyzed by chromosome counting. The p value was calculated using the unpaired t test.

To determine its association with human cancers, we examined publicly available microarray studies for dysregulation of USP44 at the mRNA level using the Oncomine database (www.Oncomine.org). Comparing cancer to normal tissues, eight cancer groups have significant alteration in the level of USP44 (Fig. 6A). In seven, the level of USP44 is reduced in the tumors compared with normal tissues. In contrast, T-cell acute lymphoblastic leukemia (T-ALL) was observed to have higher levels of USP44 mRNA. Since these studies compare T-ALL to normal bone marrow as opposed to T-cells, it is not clear if the higher expression is the result of gene dysregulation, or merely reflects high levels of USP44 in T-cells. We therefore used TaqMan-based qRT-PCR to compare the level of USP44 mRNA in a panel of 24 primary T-ALL samples to those seen in primary T-lymphocytes purified from the peripheral blood of healthy human donors. In agreement with the microarray studies, we observed a substantial increase in USP44 mRNA in T-ALL samples (Fig. 6B). In 18 out of 24 specimens, there was at least a>3-fold increase in USP44 mRNA compared with controls. The mean increase was 16-fold with a range of -4 to +63. To verify that high levels of USP44 also lead to CIN in human cells, we transduced primary human foreskin fibroblasts (HFF) with either empty or USP44 (human) encoding lentivirus followed by chromosome counts. As we observed in MEFs, USP44 over-expression led to a significant increase in aneuploidy in HFFs compared with controls (Fig. 6C). We conclude that USP44 levels are increased in T-ALL and that USP44-induced CIN may contribute to the pathogenesis of this disease.

## Discussion

Targeted ubiquitination plays an essential role in the orderly progression of cells through mitosis. Until recently, nothing was known regarding the role of de-ubiquitinating enzymes as regulators of this process. The data presented here provide three pieces of evidence that strongly support an important role for USP44 in regulating mitotic events. First, high levels of Usp44 led to significant errors in chromosome segregation resulting in cellular aneuploidy. This is a novel finding and provides compelling evidence that levels of USP44 must be tightly regulated in order to prevent errors. Second, we find that excess Usp44 results in a reinforcement of the mitotic checkpoint as measured by the ability of cells to maintain an arrest in the presence of unattached chromosome kinetochores, and by a lengthening of the time required by cells to complete mitosis. Thirdly, we find that the Cdc20-APC/C substrate cyclin B1 is stabilized by high levels of Usp44. Surprisingly, this effect was greatest in G2 rather than in mitosis. Taken together, these data provide further evidence supporting the proposed role of USP44 as a regulator of Cdc20-APC/C and the mitotic checkpoint.

USP44 has been identified in two recent functional genomic screens as an important protein in the mitotic checkpoint [14], [15]. The proposed mechanism for this activity involves antagonistic activities of the Ub E2 conjugating enzyme UbcH10 and USP44 in which UbcH10 promotes checkpoint silencing, and USP44 promotes checkpoint maintenance. Recent work has questioned this model, however, through use of a lysine-less mutant of Cdc20 [17]. In the context of the above model, this mutant would be expected to arrest cells in mitosis, as the MCC remains associated with Cdc20 due to the lack of the ubiquitin-mediated dissociation signal. This mutant was able to efficiently activate APC/C activity, bind to the MCC components BubR1 and Mad2, and was able to *dissociate* from these components upon checkpoint inactivation, suggesting that Cdc20 ubiquitination is not required for checkpoint silencing. Surprisingly, expression of this construct led to the inability of cells to maintain mitotic arrest in the presence of unattached kinetochores. This suggests that Cdc20 may itself be rate limiting in the activation of APC/C. Questions remain, however, as this effect was not strictly related to the level of Cdc20 in these experiments, suggesting that Cdc20 ubiquitination may be important for some aspects of APC/C activity. Our data support a role for Usp44 in delaying anaphase onset. In light of its previously demonstrated ability to remove ubiquitin from Cdc20, this suggests that enforced Cdc20 de-ubiquitination prevents activation of APC/C, at least in G2. It is possible, that under physiological conditions, that Cdc20 ubiquitination both affects is turnover and its association with the MCC, such that Cdc20 driven APC/C activity has a built in termination method (Cdc20 degradation). Furthermore, non-degradative ubiquitination may affect the Cdc20 functioning.

The observation that Usp44 overexpression specifically leads to anaphase bridges argues against a non-specific disruption of normal mitotic functions. We do not observe significant increases in the rates of lagging or misaligned chromosomes in cells expressing excess Usp44. The latter two defects are commonly seen in cells experiencing a bypass of the mitotic checkpoint and in the presence of defects in the microtubule –kinetochore “error correction machinery” that requires aurora B. An association has been proposed between defects in the DNA repair or replication processes and the formation of anaphase bridges [25], [26], [27]. The high levels of exogenous Usp44 in G1/S phases in our system raise the intriguing idea that the bridges observed in our experiments may be in part due to defects incurred during these S phase processes.

The idea that prolonged checkpoint signaling may lead to chromosome missegregation and aneuploidy was recently explored in the work by the Benezra group who demonstrated aneuploidy and enhanced tumorigenesis in a mouse model with an inducible Mad2 transgene [28]. These mice had a significant incidence of spontaneous tumors beginning after 12 months of age consisting of a wide variety of tumor types. Cells from these mice had prolonged mitosis, stabilized cyclin B, and had increased numbers of anaphase bridges. Our results are in line with these in that we observe an accumulation of Mad2 on Cdc20, a lengthening of mitosis, and aneuploidy. What is not clear from either of our studies, however, is the molecular mechanism linking enhanced APC/C inhibition with chromosome missegregation. It is relatively easy to envision how a weakened checkpoint would lead to chromosome missegregation as cells enter anaphase prior to full chromosome alignment. The situation here, with a strengthened checkpoint, is not as clear.

We were particularly surprised to find that the highest levels of exogenous Usp44 occurred in interphase, and that levels were relatively low in mitosis. As we clearly observe mitotic phenotypes resulting from excess Usp44, this suggests that either the levels of exogenous Usp44 are still above normal in mitosis, or that the impact of high levels in interphase persists into mitosis, leading to mitotic errors. While we clearly demonstrate that levels of cyclin B are increased in G2, consistent with the latter explanation, we cannot determine whether the levels of Usp44 are in fact elevated in mitosis due to the lack of a suitable antibody. Our findings in this regard are at odds with a recent report where high levels of Usp44-HA were seen in mitosis, with degradation coinciding with mitotic exit. While we initially thought this was due to inter-species variation, our results in HeLa cells were similar to the mouse data. More detailed work regarding the regulated degradation of USP44 is needed to clarify these discrepancies. Our work is consistent with a recent study using MEFs with low levels of BubR1 that demonstrate a requirement for APC/C inhibition in G2. While low levels of cyclin B1/Cdk activity in mitosis had previously been shown in these BubR1 hypomorphic cells [29], a report recently found that the reduced levels of cyclin B1 exist prior to mitotic onset in G2 [30]. This suggests that even prior to the formation of chromosome kinetochores, that APC/C activity is restrained to facilitate mitotic entry. While our data suggest a similar role for USP44, it will be also important to determine whether interphase levels of USP44 support this role for the endogenous enzyme.

Mutations in several CIN genes have been described in a variety of human cancers. While the majority of cases of most types of tumors have obvious aneuploidy, it is becoming more clear that, with the emerging recognition of acquired uniparental disomy, the true number of cases where aneuploidy contributes to cancer pathogenesis is likely even larger. The role of aneuploidy in T-ALL was highlighted in recent studies of the TLX1 oncogene. This gene is involved in translocations in up to 30% of adult cases of T-ALL. Recently, its role in the pathogenesis of this disease was shown to involve chromosome instability, as TLX transgenic mice developed a high incidence of T-ALL characterized by increased aneuploidy [31]. It therefore seems feasible that high levels of USP44 may contribute to the pathogenesis of T-ALL through a mechanism involving aneuploidy.

It is important to note that UbcH10 over-expression has been observed in many tumors of a variety of types. Recently, transgenic over-expression of UbcH10 was shown to drive the development of spontaneous tumors in mice, providing conclusive evidence of its oncogenic potential [32]. The proposed antagonistic relationship between this enzyme and USP44 raise the possibility that alterations in the ubiquitination status of Cdc20-APC/C or its substrates may be a recurring theme in a number of cancers.

## Materials and Methods

### Ethics Statement

The use of human disease samples was performed following the approval of the Mayo Clinic Institutional Review Board (IRB). Clinical samples were obtained following an additional review by the Children's Oncology Group (COG), Acute Lymphoblastic Leukemia Biology Subcommittee. Samples were acquired, following informed consent, by the COG in the course of several multi-institutional clinical trials, and were completely de-identified prior to their being provided to the authors. As the consent used by the COG included the use of these samples in future biologic research, the Mayo Clinic IRB waived the need for further consent prior to the use of these samples in our studies. All studies involving animals were reviewed and approved by the Mayo Clinic Institutional Animal Use and Care Committee (Protocol # A25510).

### Cloning, lentivirus production, and cell culture

Upon review of the NCBI data for mouse Usp44, we noted that its sequence and genomic structure differ from all other known alleles for this gene regardless of species. The predicted amino acid sequence from this locus would lack a catalytic cysteine and would have relatively poor alignment with the human sequence (alignment score 61; ClustalW2 (http://www.ebi.ac.uk/Tools/msa/clustalw2/)). Using the human USP44 cDNA as a benchmark, we performed a BLAT search (http://genome.ucsc.edu/) against the murine genome and identified an open reading frame with a high degree of homology to the human sequence (alignment score 78; ClustalW2) including a highly conserved catalytic cysteine. This sequence (herein referred to as Usp44) was cloned by PCR from cDNA generated from mouse kidney RNA using the RNeasy kit (Qiagen) according to manufacturer instructions. This cDNA is expressed in all tested tissues in the mouse using quantitative real-time PCR. The RNA was converted to cDNA using the superscript III kit (Invitrogen) according to manufacturer instructions. The cDNA was cloned with an N- or C-terminal HA tag, or mCherry C-terminal fusion, into the lentiviral vector pTSIN-IRES-Puro in which we inserted the IRES-puromycin resistance cassette cloned from pIRES-Puro (Clontech) into an engineered multiple cloning site that was inserted between the BamH1 and KpnI sites of pTSIN. Cells used in this study include murine embryonic fibroblasts (MEFs) that were generated from day 13.5 embryos resulting from an intercross of C57BL/6 mice [29]. Some experiments were performed with MEFS that were immortalized by transduction with the SV-40 large T antigen [33]. The results of these experiments were additionally verified in low-passage primary MEFs. All MEFs were cultured as described [32]. For all experiments, at least three independent MEF lines were used. Chromosome counts on MEFs were performed as previously described [34], [35]. Spectral karyotypic analysis was performed by the Mayo Clinic Cytogenetics Core Facility, using the protocol, reagents, instrumentation and software from Applied Spectral Imaging. Cell synchronization was performed as described [36]. Quantitation of cyclin-B1-cerulean was performed as described [30]. Prism software (GraphPad Software) was used for the generation of all graphs and statistical analysis.

### Immunoblotting and immunoprecipitation

Immunoblotting was performed using standard procedures. Immunoprecipitation was performed as described [37]. Antibodies against the following antigens were used for either immunoblotting, immunoprecipitation, or immunofluorescence; HA (3F10; Roche), β-actin (Sigma, AC-151), phospho histone H3(Ser10) (Upstate, 06-570), cyclin B1 (Santa Cruz, SC-245), Cdc20 (Santa Cruz, SC-8358), Cdc27 (BD Biosciences, 610454), BubR1 [29], Mad2 (BD Biosciences, 610678), cyclin A (Santa Cruz, ), securin (Abcam), human anticentromere antibody (Antibodies, Inc.).

### Immunofluorescent and fluorescent live-cell microscopy

Live cell imaging experiments were performed as previously described in detail [20], [36]. A lentiviral construct encoding YFP-tagged H2B (pTSIN-H2B-YFP) was used to visualize of chromosomes by fluorescence microscopy. Indirect immunofluorescence was performed as described [37], [38]. A laser-scanning microscope (LSM 510 v3.2SP2; Carl Zeiss, Inc.) with Axiovert 100 M (Carl Zeiss, Inc.) with a c-Apochromat 100x oil immersion objective was used to analyze immunostained cells and capture representative images. For quantification of cyclin-B1^Cerulean^ and Usp44^Cherry^ levels, at each mitotic stage, 10 or more cells were analyzed per each of 3 MEF lines. The mean fluorescence intensity was determined after background subtraction of images transformed to 8-bit grayscale using Image-J software (National Institutes of Health).

### Analysis of T-cell leukemia

Following approval by the Mayo Clinic Institutional Review Board (IRB), a series of 25 de-identified samples with a diagnosis of T-cell acute lymphoblastic leukemia (ALL) were obtained from Mayo Clinic sample banks, and from the Children's Oncology Group (with approval of the ALL committee), and were used for analysis of USP44 mRNA. Controls consisted of peripheral T-cells isolated from discarded filter-cones from volunteer donors to the Mayo Clinic blood donor center [39]. RNA was extracted from single cell suspensions as described [40] and was converted to cDNA using the superscript III system (Invitrogen) according to manufacturer instructions. Quantitative real-time PCR was performed with TaqMan probes Hs500259765_m1 USP44 and Hu ACTB-FAM-MGB. All experiments were performed in triplicate and USP44 mRNA data were normalized to β-actin (Hu ACTB-FAM-MGB). Fold-change compared with controls was calculated with the delta-delta Ct method.
